# Crystal structure of *fac*-tri­carbonyl­chlorido­bis­(4-hy­droxy­pyridine)­rhenium(I)–pyridin-4(1*H*)-one (1/1)

**DOI:** 10.1107/S2056989017013512

**Published:** 2017-09-29

**Authors:** Saray Argibay-Otero, Rosa Carballo, Ezequiel M. Vázquez-López

**Affiliations:** a Departamento de Química Inorgánica, Facultade de Química, Instituto de Investigación Sanitaria Galicia Sur – Universidade de Vigo, Campus Universitario, E-36310 Vigo, Galicia, Spain

**Keywords:** crystal structure, rhenium(I) compounds, 4-hy­droxy­pyridine, pyridin-4(1*H*)-one

## Abstract

The asymmetric unit of the title compound, [ReCl(C_5_H_5_NO)_2_(CO)_3_]·C_5_H_5_NO, contains one mol­ecule of the complex *fac*-[ReCl(4-pyOH)_2_(CO)_3_] (where 4-pyOH represents 4-hy­droxy­pyridine) and one mol­ecule of pyridin-4(1*H*)-one (4-HpyO). In the mol­ecule of the complex, the Re atom is coordinated to two N atoms of the two 4-pyOH ligands, three carbonyl C atoms, in a facial configuration, and the Cl atom. The resulting geometry is slightly distorted octa­hedral. In the crystal structure, both fragments are associated by hydrogen bonds; two 4-HpyO mol­ecules bridge between two mol­ecules of the complex using the O=C group as acceptor for two different HO– groups of coordinated 4-pyOH from two neighbouring metal complexes. The resulting square arrangements are extented into infinite chains by hydrogen bonds involving the N—H groups of the 4-HpyO mol­ecule and the chloride ligands. The chains are further stabilized by π-stacking inter­actions.

## Abstract 2

Mol­ecules of the complex *fac*-[ReCl(4-pyOH)_2_(CO)_3_] (4-pyOH is 4-hy­droxy­pyridine) and 4-pyridone are associated in ladder chains by hydrogen and coordination bonds.

## Chemical context   

The structural stability of the *fac*-{Re^I^(CO)_3_} fragment and its trend to form sixfold coordinated octa­hedral complexes make it a suitable candidate for the construction of self-assambled metallomacrocycles, with some of them showing inter­esting properties (Slone *et al.*, 1998 ▸; Sun & Lees, 2002 ▸). Bi­pyridine (and pyrazine) based ligands are usually chosen to obtain square or rectangular metallocycles, [Re_4_(*L*)_4_(CO)_12_] (*L* is the bridging ligand) with inter­nal diameters of 5–9 nm. In the present work, we present the structure of a rhenium complex, where the square architecture is achieved by a coordinative Re—*L* link (where *L* is 4-hy­droxy­pyridine) and by hydrogen-bonding inter­actions involving a 4-pyridone mol­ecule (a tautomer of 4-hy­droxy­pyridine *L*).
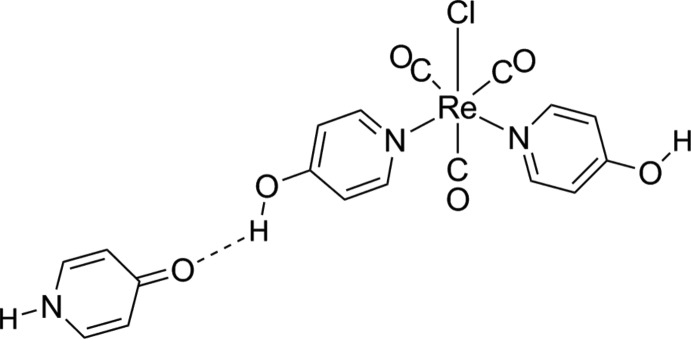


## Structural commentary   

**Figure 1:**
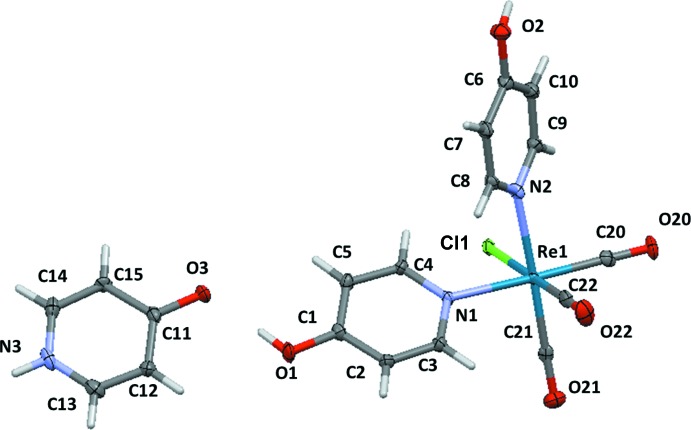
The mol­ecular structure of the title compound, with displacement ellipsoids drawn at the 50% probability level.

The crystal structure consists of mol­ecules of *fac*-[ReCl(4-pyOH)_2_(CO)_3_] (where 4-pyOH represents 4-hy­droxy­pyridine) and pyridin-4(1*H*)-one (4-HpyO) in a 1:1 ratio (Fig. 1 ▸). Both mol­ecules are associated through hydrogen bonding (see below). The existence of the pyridone form instead of hy­droxy­pyridine is confirmed by the C—O bond distance, subtanti­ally shorthened in 4-HpyO [C11—O3 = 1.293 (5) Å] with respect to the coordinated 4-pyOH [O1—C1 = 1.335 (5) Å and O2—C6 = 1.339 (5) Å], indicating the presence of a double C=O bond in 4-HpyO. The C—C bond lengths involving the carbonyl group [C11—C12 = 1.425 (6) Å and C11—C15 = 1.432 (6) Å] are elongated with respect to those observed in the 4-pyOH fragments [for instance, C1—C2 = 1.404 (6) Å and C1—C5 = 1.395 (6) Å]. The C—N bond lengths are also longer than their typical values in pyridines or pyridinium cations. These parameters are close to those found in the crystal structure of the free (uncoordinated) 4-pyridone (Jones, 2001 ▸; Tyl *et al.*, 2008 ▸) or to those involved in hydrogen bonding (Campos-Gaxiola *et al.* 2014 ▸; Staun & Oliver, 2012 ▸; 2015 ▸).

The mol­ecular structure of *fac*-[ReCl(4-pyOH)_2_(CO)_3_] is similar to other tri­carbonyl­rhenium(I) complexes with two pyridine-based ligands (Abel & Wilkinson, 1959 ▸; Farrell *et al.*, 2016 ▸). The coordination polyhedron around the Re atom can be described as slightly distorted octa­hedral (all angles are close to 90 or 180°), formed by coordination of the two N atoms of the two 4-pyOH ligands (N1 and N2), by the three carbonyl C atoms, in a facial configuration, and the chloride ligand. Both Re—N bond lengths [2.208 (4) and 2.210 (4) Å] are statistically equivalent. Neverthless, the Re—Cl bond in the present compound [2.4986 (10) Å] is longer that those found in pyridine derivatives described recently by Farrell *et al.* (2016 ▸), with an average value of 2.4649 (4) Å. This fact is likely due to the hydrogen-bonding inter­action involving the chloride and the N—H group of a neighbouring 4-pyridone since this inter­action is absent in those structures.

## Supra­molecular features   

**Figure 2:**
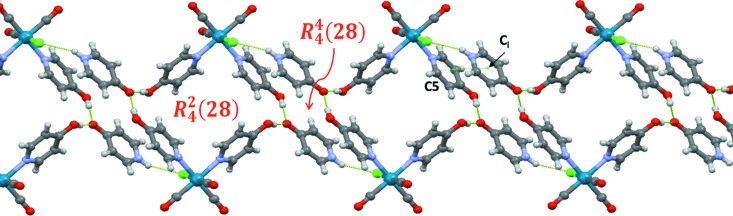
Representation of the formation of chains by hydrogen-bonding and π-stacking in the crystal structure.

**Figure 3:**
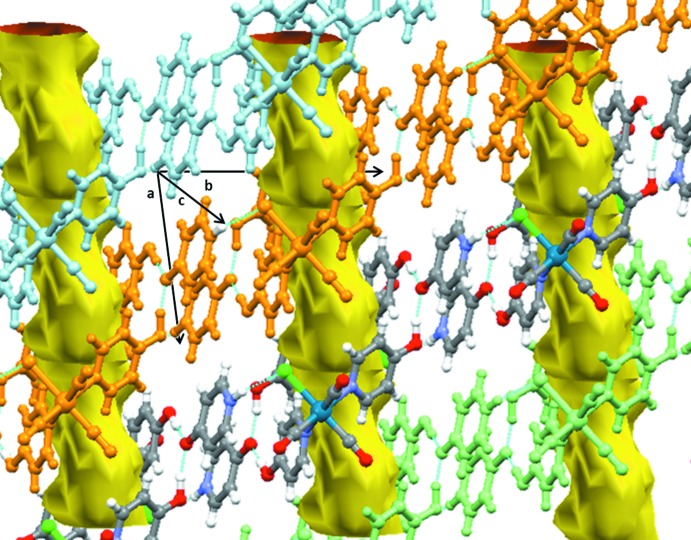
Association of the chains and formation of the empty channels in the crystal structure.

The mol­ecular association in the crystal is strongly directed by hydrogen bonding (Table 1 ▸). Two 4-pyridone mol­ecules bridge between two *fac*-[ReCl(4-pyOH)_2_(CO)_3_] using the ketone O=C group as the hydrogen-bonding acceptor to two different HO– groups, forming 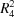
(28) rings centred at the *g* Wyckoff site (Fig. 2 ▸). The N—H group of the pyridone unit also establishes hydrogen-bond inter­actions, with the chloride group, yielding a new centrosymmetric ring 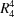
(28) (at the *f* Wickoff site). Although the centroid-to-centroid distance between the pyridone and hy­droxy­pyridone is rather long (3.791 Å), some distances between the atoms and centroids of the rings [C4⋯N3^vi^ = 3.231 Å, C4⋯C14^vi^ = 3.470 Å, C5⋯C14^vi^ = 3.478 Å and C5⋯Ci^vi^ = 3.365 Å; symmetry code: (vi) 1 − *x*, 2 − *y*, 1 − *z*; see Fig. 2 ▸] suggest a (slipped) π-stacking inter­action. Both inter­molecular inter­actions work to form infinite chains, as represented in Fig. 2 ▸, which are further supported by weak C—H⋯O and C—H⋯Cl inter­actions (the most representative ones are included in Table 1 ▸). The formation of the 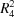
(28) rings yields a small channel-like void of *ca* 103 Å^3^ per unit cell, as shown in Fig. 3 ▸. No substantial electron density is found in the channels (*ca* 4 electrons per void based on a *PLATON*/SQUEEZE analysis (Spek, 2009 ▸, 2015 ▸).

## Database survey   

The structures of several complexes with the metal centre *fac*-tri­carbonyl­rhenium(I) and pyridine-based ligands have been reported (Abel & Wilkinson, 1959 ▸; Farrell *et al.*, 2016 ▸). The pyridine fragment can be part of a bridging ligand between different metal centres to form tetra­nuclear complexes as reported by Levine *et al.* (2009 ▸). When ligands based on 4,4′-bi­pyridine are chosen, square (Slone *et al.*, 1996 ▸; Bera *et al.*, 2004 ▸; Sun *et al.*, 2002 ▸) or rectangular (Dinolfo & Hupp, 2004 ▸; Gupta *et al.*, 2011 ▸; Lu *et al.*, 2012 ▸; Nagarajaprakash *et al.*, 2014 ▸; Orsa *et al.*, 2007 ▸) homo- or heteronuclear complexes are isolated. Applications of these compounds as sensors (Keefe *et al.*, 2000 ▸), luminescent materials (Slone *et al.*, 1996 ▸) or cytotoxic agents (Orsa *et al.*, 2007 ▸) have been also reported.

## Synthesis and crystallization   

The complex *fac*-[ReCl(4-pyOH)_2_(CO)_3_] was obtained by refluxing for 90 min a mixture of 4-hy­droxy­pyridine (29 mg, 0.31 mmol) and [ReCl(CH_3_CN)_2_(CO)_3_] in chloro­form–methanol (1:1 *v*/*v*, 10 ml). The solution was concentrated (to half of initial volume), diethyl ether was added and the mixture cooled to 277 K. Finally, the solid was filtered off and vacuum dried on CaCl_2_ (yield: 81%, 30 mg; m.p. 418–421 K). Analysis, calculated for C_13_H_10_ClN_2_O_5_Re: C 31.5, H 2.0, N 5.6%; found: C 31.9, H 1.9, N 5.5%. MS–ESI [*m*/*z* (%)]: 461 (100) [*M* – Cl]^+^. IR (ATR, cm^−1^): 2016 (*m*), 1865 (*b*, *s*), ν(CO).

Single crystals of the title compound (too few for elemental analysis or meaningful estimation of the yield) were obtained from solutions of *fac*-[ReCl(CO)_3_(4-pyOH)_2_] in CHCl_3_:CH_2_Cl_2_:ether (1:1:1) stored at 253 K (several days).

## Refinement   

Crystal data, data collection and structure refinement details are summarized in Table 2 ▸. H atoms on O and N atoms were located *via* difference Fourier analyses and refined with *U*_iso_(H) = 1.5*U*_eq_(O) and 1.2*U*_eq_(N). Other H atoms were included at calculated sites and allowed to ride on their carrier atoms, with *U*_iso_(H) = 1.2*U*_eq_(C).

## Supplementary Material

Crystal structure: contains datablock(s) I, global. DOI: 10.1107/S2056989017013512/zl2716sup1.cif

Structure factors: contains datablock(s) I. DOI: 10.1107/S2056989017013512/zl2716Isup2.hkl

Supporting information file. DOI: 10.1107/S2056989017013512/zl2716Isup3.cdx

CCDC reference: 1575682

Additional supporting information:  crystallographic information; 3D view; checkCIF report

## Figures and Tables

**Table 1:** Hydrogen-bond geometry (Å, °)

*D*—H⋯*A*	*D*—H	H⋯*A*	*D*⋯*A*	*D*—H⋯*A*
O1—H1⋯O3	0.94 (8)	1.62 (8)	2.556 (5)	175 (7)
O2—H2⋯O3^i^	1.01 (7)	1.57 (7)	2.569 (5)	169 (6)
N3—H3*A*⋯Cl1^ii^	0.97 (7)	2.32 (7)	3.218 (5)	152 (5)
C9—H9⋯O22^iii^	0.95	2.56	3.317 (7)	137
C3—H3⋯Cl1^iv^	0.95	2.89	3.580 (5)	131
C14—H14⋯O21^v^	0.95	2.62	3.264 (7)	126

Symmetry codes: (i) 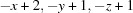; (ii) 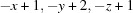; (iii) 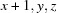; (iv) 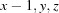; (v) 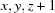.

**Table 2:** Experimental details

Crystal data	
Chemical formula	[ReCl(C_5_H_5_NO)_2_(CO)_3_]·C_5_H_5_NO
*M*_r_	590.98
Crystal system, space group	Triclinic, *P* 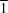
Temperature (K)	100
*a*, *b*, *c* (Å)	7.5235 (13), 11.717 (2), 13.644 (2)
α, β, γ (°)	66.694 (4), 78.757 (4), 81.374 (4)
*V* (Å^3^)	1079.9 (3)
*Z*	2
Radiation type	Mo *K*α
μ (mm^−1^)	5.79
Crystal size (mm)	0.36 × 0.35 × 0.04

Data collection	
Diffractometer	Bruker D8 Venture Photon 100 CMOS
Absorption correction	Multi-scan (*SADABS*; Krause *et al.*, 2015 ▸)
*T*_min_, *T*_max_	0.352, 0.647
No. of measured, independent and observed [*I* > 2σ(*I*)] reflections	28225, 4476, 4312
*R*_int_	0.041
(sin θ/λ)_max_ (Å^−1^)	0.630

Refinement	
*R*[*F* ^2^ > 2σ(*F* ^2^)], *wR*(*F* ^2^), *S*	0.029, 0.077, 1.35
No. of reflections	4476
No. of parameters	272
H-atom treatment	H atoms treated by a mixture of independent and constrained refinement
Δρ_max_, Δρ_min_ (e Å^−3^)	1.63, −1.03

Computer programs: *APEX3* (Bruker, 2014 ▸), *SAINT* (Bruker, 2014 ▸), *SHELXS2013* (Sheldrick, 2015*a* ▸), *SHELXL2013* (Sheldrick, 2015*b* ▸).

## supplementary crystallographic information

### Crystal data

**Table:**

[ReCl(C_5_H_5_NO)_2_(CO)_3_]·C_5_H_5_NO	*F*(000) = 568
*M**_r_* = 590.98	*D*_x_ = 1.818 Mg m^−^^3^
Triclinic, *P*1	Melting point: 145 K
*a* = 7.5235 (13) Å	Mo *K*α radiation, λ = 0.71073 Å
*b* = 11.717 (2) Å	Cell parameters from 9858 reflections
*c* = 13.644 (2) Å	θ = 3.3–26.6°
α = 66.694 (4)°	µ = 5.79 mm^−^^1^
β = 78.757 (4)°	*T* = 100 K
γ = 81.374 (4)°	Plate, yellow
*V* = 1079.9 (3) Å^3^	0.36 × 0.35 × 0.04 mm
*Z* = 2	

### Data collection

**Table:**

Bruker D8 Venture Photon 100 CMOS diffractometer	4312 reflections with *I* > 2σ(*I*)
φ and ω scans	*R*_int_ = 0.041
Absorption correction: multi-scan (SADABS; Krause *et al.*, 2015)	θ_max_ = 26.6°, θ_min_ = 2.8°
*T*_min_ = 0.352, *T*_max_ = 0.647	*h* = −9→9
28225 measured reflections	*k* = −14→14
4476 independent reflections	*l* = −17→17

### Refinement

**Table:**

Refinement on *F*^2^	Secondary atom site location: difference Fourier map
Least-squares matrix: full	Hydrogen site location: mixed
*R*[*F*^2^ > 2σ(*F*^2^)] = 0.029	H atoms treated by a mixture of independent and constrained refinement
*wR*(*F*^2^) = 0.077	*w* = 1/[σ^2^(*F*_o_^2^) + (0.0178*P*)^2^ + 5.8451*P*] where *P* = (*F*_o_^2^ + 2*F*_c_^2^)/3
*S* = 1.35	(Δ/σ)_max_ = 0.001
4476 reflections	Δρ_max_ = 1.63 e Å^−^^3^
272 parameters	Δρ_min_ = −1.03 e Å^−^^3^
0 restraints	Extinction correction: SHELXL2013 (Sheldrick, 2015b), Fc^*^=kFc[1+0.001xFc^2^λ^3^/sin(2θ)]^-1/4^
Primary atom site location: structure-invariant direct methods	Extinction coefficient: 0.0119 (9)

### Special details

**Table:**

Geometry. All esds (except the esd in the dihedral angle between two l.s. planes) are estimated using the full covariance matrix. The cell esds are taken into account individually in the estimation of esds in distances, angles and torsion angles; correlations between esds in cell parameters are only used when they are defined by crystal symmetry. An approximate (isotropic) treatment of cell esds is used for estimating esds involving l.s. planes.

### Fractional atomic coordinates and isotropic or equivalent isotropic displacement parameters (Å^2^)

**Table:**

	*x*	*y*	*z*	*U*_iso_*/*U*_eq_	
Re1	0.86373 (2)	0.70746 (2)	0.07222 (2)	0.01123 (10)	
Cl1	1.09377 (15)	0.82393 (10)	0.09609 (9)	0.0135 (2)	
O1	0.3391 (5)	0.8384 (4)	0.4444 (3)	0.0208 (8)	
H1	0.394 (10)	0.843 (6)	0.499 (6)	0.031*	
O2	1.1814 (5)	0.2036 (3)	0.4087 (3)	0.0229 (8)	
O3	0.4725 (5)	0.8444 (3)	0.6006 (3)	0.0203 (8)	
H2	1.319 (10)	0.196 (6)	0.399 (6)	0.030*	
O20	1.1184 (5)	0.6629 (4)	−0.1173 (3)	0.0232 (8)	
O21	0.6852 (5)	0.9364 (4)	−0.0929 (3)	0.0243 (8)	
O22	0.5800 (6)	0.5598 (4)	0.0526 (4)	0.0299 (9)	
N1	0.6881 (5)	0.7397 (4)	0.2102 (3)	0.0129 (8)	
N2	0.9814 (5)	0.5408 (4)	0.1954 (3)	0.0138 (8)	
N3	0.1310 (6)	0.9766 (4)	0.8090 (4)	0.0225 (9)	
H3A	0.056 (9)	1.011 (6)	0.859 (6)	0.027*	
C1	0.4552 (7)	0.8016 (4)	0.3733 (4)	0.0154 (9)	
C2	0.3875 (7)	0.7932 (5)	0.2880 (4)	0.0165 (10)	
H2A	0.2607	0.8072	0.2848	0.020*	
C3	0.5057 (7)	0.7646 (4)	0.2088 (4)	0.0151 (9)	
H3	0.4577	0.7621	0.1503	0.018*	
C4	0.7506 (7)	0.7409 (4)	0.2958 (4)	0.0140 (9)	
H4	0.8767	0.7204	0.3000	0.017*	
C5	0.6415 (7)	0.7705 (5)	0.3779 (4)	0.0166 (10)	
H5	0.6922	0.7697	0.4368	0.020*	
C6	1.1246 (7)	0.3148 (4)	0.3390 (4)	0.0169 (10)	
C7	0.9392 (7)	0.3537 (5)	0.3529 (4)	0.0187 (10)	
H7	0.8585	0.3038	0.4120	0.022*	
C8	0.8757 (7)	0.4642 (4)	0.2803 (4)	0.0149 (9)	
H8	0.7493	0.4884	0.2904	0.018*	
C9	1.1622 (7)	0.5051 (5)	0.1839 (4)	0.0170 (10)	
H9	1.2407	0.5590	0.1264	0.020*	
C10	1.2371 (7)	0.3934 (5)	0.2526 (4)	0.0191 (10)	
H10	1.3638	0.3706	0.2408	0.023*	
C11	0.3637 (7)	0.8866 (4)	0.6664 (4)	0.0159 (9)	
C12	0.1806 (7)	0.9321 (5)	0.6507 (4)	0.0192 (10)	
H12	0.1357	0.9326	0.5901	0.023*	
C13	0.0699 (7)	0.9751 (5)	0.7227 (4)	0.0206 (10)	
H13	−0.0523	1.0045	0.7120	0.025*	
C14	0.3048 (8)	0.9366 (5)	0.8259 (4)	0.0202 (10)	
H14	0.3458	0.9412	0.8857	0.024*	
C15	0.4216 (6)	0.8902 (4)	0.7590 (4)	0.0149 (9)	
H15	0.5419	0.8601	0.7736	0.018*	
C20	1.0245 (7)	0.6793 (4)	−0.0466 (4)	0.0175 (10)	
C21	0.7553 (6)	0.8525 (4)	−0.0305 (4)	0.0148 (9)	
C22	0.6898 (7)	0.6144 (5)	0.0594 (4)	0.0197 (10)	

### Atomic displacement parameters (Å^2^)

**Table:**

	*U*^11^	*U*^22^	*U*^33^	*U*^12^	*U*^13^	*U*^23^
Re1	0.01149 (13)	0.01337 (13)	0.01111 (13)	0.00016 (7)	−0.00161 (7)	−0.00747 (8)
Cl1	0.0141 (5)	0.0153 (5)	0.0144 (5)	−0.0007 (4)	−0.0023 (4)	−0.0091 (4)
O1	0.0169 (18)	0.032 (2)	0.0186 (18)	0.0003 (15)	0.0016 (15)	−0.0177 (16)
O2	0.024 (2)	0.0183 (18)	0.0187 (18)	0.0032 (15)	−0.0025 (15)	−0.0015 (15)
O3	0.0179 (18)	0.0264 (19)	0.0191 (18)	0.0058 (15)	−0.0019 (14)	−0.0144 (15)
O20	0.026 (2)	0.0278 (19)	0.0184 (18)	−0.0017 (16)	0.0063 (16)	−0.0159 (16)
O21	0.025 (2)	0.0226 (19)	0.0214 (19)	0.0016 (16)	−0.0089 (16)	−0.0037 (16)
O22	0.024 (2)	0.037 (2)	0.040 (2)	−0.0070 (17)	−0.0022 (18)	−0.026 (2)
N1	0.0115 (19)	0.0162 (19)	0.0112 (18)	0.0018 (15)	0.0003 (15)	−0.0072 (15)
N2	0.0117 (19)	0.0133 (18)	0.016 (2)	−0.0008 (15)	0.0001 (16)	−0.0066 (16)
N3	0.024 (2)	0.020 (2)	0.023 (2)	−0.0028 (18)	0.0078 (19)	−0.0120 (18)
C1	0.014 (2)	0.017 (2)	0.015 (2)	−0.0020 (18)	0.0015 (18)	−0.0073 (18)
C2	0.016 (2)	0.020 (2)	0.017 (2)	0.0035 (18)	−0.0061 (19)	−0.0097 (19)
C3	0.015 (2)	0.017 (2)	0.016 (2)	−0.0007 (18)	−0.0037 (18)	−0.0090 (19)
C4	0.017 (2)	0.016 (2)	0.012 (2)	0.0009 (18)	−0.0043 (18)	−0.0074 (18)
C5	0.018 (2)	0.019 (2)	0.016 (2)	−0.0022 (19)	−0.0033 (19)	−0.0102 (19)
C6	0.020 (2)	0.014 (2)	0.017 (2)	0.0006 (18)	−0.0036 (19)	−0.0059 (19)
C7	0.019 (2)	0.016 (2)	0.017 (2)	0.0008 (19)	0.002 (2)	−0.0053 (19)
C8	0.013 (2)	0.018 (2)	0.015 (2)	−0.0006 (18)	0.0007 (18)	−0.0089 (19)
C9	0.015 (2)	0.020 (2)	0.016 (2)	−0.0006 (18)	−0.0006 (19)	−0.0077 (19)
C10	0.015 (2)	0.021 (2)	0.020 (2)	0.0025 (19)	−0.002 (2)	−0.008 (2)
C11	0.021 (2)	0.014 (2)	0.013 (2)	0.0001 (18)	−0.0003 (19)	−0.0074 (18)
C12	0.022 (3)	0.019 (2)	0.017 (2)	0.005 (2)	−0.005 (2)	−0.009 (2)
C13	0.018 (2)	0.018 (2)	0.027 (3)	0.0001 (19)	−0.001 (2)	−0.012 (2)
C14	0.027 (3)	0.021 (2)	0.017 (2)	−0.007 (2)	0.000 (2)	−0.011 (2)
C15	0.011 (2)	0.016 (2)	0.022 (2)	−0.0007 (17)	−0.0068 (19)	−0.0106 (19)
C20	0.021 (3)	0.011 (2)	0.024 (3)	0.0000 (18)	−0.008 (2)	−0.0085 (19)
C21	0.010 (2)	0.019 (2)	0.017 (2)	−0.0061 (18)	0.0021 (18)	−0.0086 (19)
C22	0.014 (2)	0.027 (3)	0.016 (2)	0.005 (2)	0.0001 (19)	−0.011 (2)

### Geometric parameters (Å, º)

**Table:**

Re1—C22	1.898 (6)	C2—C3	1.376 (7)
Re1—C21	1.914 (5)	C2—H2A	0.9500
Re1—C20	1.933 (5)	C3—H3	0.9500
Re1—N1	2.208 (4)	C4—C5	1.383 (7)
Re1—N2	2.210 (4)	C4—H4	0.9500
Re1—Cl1	2.4987 (11)	C5—H5	0.9500
O1—C1	1.333 (6)	C6—C10	1.393 (7)
O1—H1	0.94 (8)	C6—C7	1.401 (7)
O2—C6	1.341 (6)	C7—C8	1.367 (7)
O2—H2	1.01 (7)	C7—H7	0.9500
O3—C11	1.289 (6)	C8—H8	0.9500
O20—C20	1.143 (7)	C9—C10	1.387 (7)
O21—C21	1.151 (6)	C9—H9	0.9500
O22—C22	1.155 (7)	C10—H10	0.9500
N1—C4	1.348 (6)	C11—C12	1.426 (7)
N1—C3	1.362 (6)	C11—C15	1.433 (7)
N2—C8	1.346 (6)	C12—C13	1.363 (7)
N2—C9	1.360 (6)	C12—H12	0.9500
N3—C13	1.353 (7)	C13—H13	0.9500
N3—C14	1.353 (7)	C14—C15	1.355 (7)
N3—H3A	0.97 (7)	C14—H14	0.9500
C1—C2	1.401 (7)	C15—H15	0.9500
C1—C5	1.402 (7)		
			
C22—Re1—C21	87.9 (2)	C5—C4—H4	118.2
C22—Re1—C20	89.6 (2)	C4—C5—C1	119.2 (4)
C21—Re1—C20	88.6 (2)	C4—C5—H5	120.4
C22—Re1—N1	91.96 (19)	C1—C5—H5	120.4
C21—Re1—N1	92.49 (18)	O2—C6—C10	124.3 (5)
C20—Re1—N1	178.11 (17)	O2—C6—C7	117.8 (5)
C22—Re1—N2	91.80 (19)	C10—C6—C7	117.8 (4)
C21—Re1—N2	177.97 (17)	C8—C7—C6	119.2 (5)
C20—Re1—N2	93.45 (18)	C8—C7—H7	120.4
N1—Re1—N2	85.51 (15)	C6—C7—H7	120.4
C22—Re1—Cl1	177.81 (16)	N2—C8—C7	124.0 (5)
C21—Re1—Cl1	94.05 (14)	N2—C8—H8	118.0
C20—Re1—Cl1	91.33 (15)	C7—C8—H8	118.0
N1—Re1—Cl1	87.03 (11)	N2—C9—C10	122.8 (5)
N2—Re1—Cl1	86.19 (11)	N2—C9—H9	118.6
C1—O1—H1	113 (4)	C10—C9—H9	118.6
C6—O2—H2	109 (4)	C9—C10—C6	119.2 (5)
C4—N1—C3	116.7 (4)	C9—C10—H10	120.4
C4—N1—Re1	124.0 (3)	C6—C10—H10	120.4
C3—N1—Re1	119.2 (3)	O3—C11—C12	122.0 (5)
C8—N2—C9	116.8 (4)	O3—C11—C15	121.4 (5)
C8—N2—Re1	121.6 (3)	C12—C11—C15	116.6 (4)
C9—N2—Re1	121.4 (3)	C13—C12—C11	120.0 (5)
C13—N3—C14	120.9 (5)	C13—C12—H12	120.0
C13—N3—H3A	123 (4)	C11—C12—H12	120.0
C14—N3—H3A	116 (4)	N3—C13—C12	121.1 (5)
O1—C1—C2	118.1 (4)	N3—C13—H13	119.4
O1—C1—C5	124.5 (5)	C12—C13—H13	119.4
C2—C1—C5	117.4 (4)	N3—C14—C15	121.3 (5)
C3—C2—C1	119.6 (5)	N3—C14—H14	119.4
C3—C2—H2A	120.2	C15—C14—H14	119.4
C1—C2—H2A	120.2	C14—C15—C11	120.0 (5)
N1—C3—C2	123.2 (4)	C14—C15—H15	120.0
N1—C3—H3	118.4	C11—C15—H15	120.0
C2—C3—H3	118.4	O20—C20—Re1	179.5 (4)
N1—C4—C5	123.7 (5)	O21—C21—Re1	177.0 (4)
N1—C4—H4	118.2	O22—C22—Re1	178.1 (4)

### Hydrogen-bond geometry (Å, º)

**Table:**

*D*—H···*A*	*D*—H	H···*A*	*D*···*A*	*D*—H···*A*
O1—H1···O3	0.94 (8)	1.62 (8)	2.556 (5)	175 (7)
O2—H2···O3^i^	1.01 (7)	1.57 (7)	2.569 (5)	169 (6)
N3—H3*A*···Cl1^ii^	0.97 (7)	2.32 (7)	3.218 (5)	152 (5)
C9—H9···O22^iii^	0.95	2.56	3.317 (7)	137
C3—H3···Cl1^iv^	0.95	2.89	3.580 (5)	131
C14—H14···O21^v^	0.95	2.62	3.264 (7)	126

Symmetry codes: (i) −*x*+2, −*y*+1, −*z*+1; (ii) −*x*+1, −*y*+2, −*z*+1; (iii) *x*+1, *y*, *z*; (iv) *x*−1, *y*, *z*; (v) *x*, *y*, *z*+1.
